# Wearable Sensor-Based Assessments for Remotely Screening Early-Stage Parkinson’s Disease

**DOI:** 10.3390/s24175637

**Published:** 2024-08-30

**Authors:** Shane Johnson, Michalis Kantartjis, Joan Severson, Ray Dorsey, Jamie L. Adams, Tairmae Kangarloo, Melissa A. Kostrzebski, Allen Best, Michael Merickel, Dan Amato, Brian Severson, Sean Jezewski, Steve Polyak, Anna Keil, Josh Cosman, David Anderson

**Affiliations:** 1 Clinical Ink, Winston-Salem, NC 27101, USA; shane.johnson@clinicalink.com (S.J.); allen.best@clinicalink.com (A.B.); dan.jacksonamato@clinicalink.com (D.A.); brian.severson@clinicalink.com (B.S.); sean.jezewski@clinicalink.com (S.J.);; 2 Center for Health and Technology, University of Rochester Medical Center, Rochester, NY 14623, USAmelissa.kostrzebski@chet.rochester.edu (M.A.K.); 3 Department of Neurology, University of Rochester Medical Center, Rochester, NY 14623, USA; 4 Takeda Pharmaceuticals, Cambridge, MA 02142, USA; 5 AbbVie Pharmaceuticals, North Chicago, IL 60064, USA; josh.cosman@abbvie.com

**Keywords:** Parkinson’s disease, wearable sensors, digital biomarkers, remote monitoring, gait analysis, phonation, feature engineering, mobile health technologies, early detection

## Abstract

Prevalence estimates of Parkinson’s disease (PD)—the fastest-growing neurodegenerative disease—are generally underestimated due to issues surrounding diagnostic accuracy, symptomatic undiagnosed cases, suboptimal prodromal monitoring, and limited screening access. Remotely monitored wearable devices and sensors provide precise, objective, and frequent measures of motor and non-motor symptoms. Here, we used consumer-grade wearable device and sensor data from the WATCH-PD study to develop a PD screening tool aimed at eliminating the gap between patient symptoms and diagnosis. Early-stage PD patients (*n* = 82) and age-matched comparison participants (*n* = 50) completed a multidomain assessment battery during a one-year longitudinal multicenter study. Using disease- and behavior-relevant feature engineering and multivariate machine learning modeling of early-stage PD status, we developed a highly accurate (92.3%), sensitive (90.0%), and specific (100%) random forest classification model (AUC = 0.92) that performed well across environmental and platform contexts. These findings provide robust support for further exploration of consumer-grade wearable devices and sensors for global population-wide PD screening and surveillance.

## 1. Introduction

Parkinson’s disease (PD) is the fastest-growing neurological disorder, with 6.2 million individuals currently affected worldwide and 14.2 million individuals projected to be affected by 2040 [1]. PD prevalence studies are heterogeneous and often underestimate current and projected cases of PD [2,3]. Limited capabilities surrounding screening resources may contribute to underestimating the global PD burden [4].

PD is a multidomain disease that is traditionally characterized by motor symptoms via in-clinic evaluations [5], which are subjective, rater-dependent, and infrequent [6,7]. Diagnostic accuracy using these methods, particularly in early-stage PD, ranges between 58% and 85% [8,9]. Population-based studies have found that 12% to 83% of individuals presenting with PD symptoms are undiagnosed [10]. Detecting patients who are transitioning from prodromal to early-stage PD remains challenging [11]. Emerging biomarker-based approaches may have promise in this domain, but these require clinic visits and invasive spinal tap procedures that may limit their widespread adoption [12]. Thus, developing methods and capabilities that provide easier access to more efficient and accurate screening resources promises to facilitate more frequent symptom monitoring, thereby enabling earlier diagnostic and treatment interventions and overall better long-term quality of life.

Wearable devices and sensors generate information-rich continuous data streams that can measure disease- and behavior-relevant signals [13]. Digital measures engineered from wearable devices and sensor data offer opportunities to more precisely, objectively, and frequently monitor the patient’s disease burden relative to traditional clinical endpoints [14,15,16]. Numerous studies have evaluated wearable devices and sensors in PD symptom monitoring [17,18,19,20,21,22,23,24,25,26] and machine-learning capabilities are emerging to develop multivariate predictive models of PD [27]. For example, Arora and colleagues recorded smartphone sensor-based assessments of voice, balance, gait, finger tapping, and response time in a small cohort of PD patients and non-demographically matched health comparisons and found that a random forest model achieved a sensitivity and specificity of 96.2% and 96.9%, respectively, for predicting PD status [18]. Critically, however, the investigators in this study performed record-wise rather than subject-wise cross-validation, which overestimates classification performance due to the non-independence of the training and test datasets. In a separate study, Omberg and colleagues recorded smartphone sensor-based assessments of finger tapping, voice, gait, and balance in a large cohort of self-reported PD and non-PD participants in the mPower study and found that a random forest model achieved an AUC of 0.80 for predicting PD status [23]. This study was limited, however, by reduced control over enrollment screening and data quality, the use of non-demographically matched study groups, and non-clinically confirmed PD status. Existing studies, therefore, have been insufficient in capturing the multi-domain sequelae of PD, focusing instead on capturing data from a single device or sensor modality. Furthermore, despite emerging interest in using machine learning capabilities to develop multivariate predictive models of PD [27], large-scale studies of wearable devices and sensors used by clinically confirmed PD patients have yet to develop a robust classification model of PD status that can be implemented globally as a widely accessible screening tool.

In the current work, our goal was to develop a PD screening tool using consumer-grade wearable devices and sensors. To this end, we implemented feature engineering and multivariate machine learning modeling using data generated from the large-scale multicenter WATCH-PD (Wearable Assessments in the Clinic and at Home in PD) study (NCT03681015). Briefly, WATCH-PD was a one-year longitudinal study of clinically confirmed early-stage PD and age-matched comparison group (HC) participants who completed a multidomain (i.e., cognitive, psychomotor, speech, and mobility) battery of assessments that acquired data from multiple sensors equipped on consumer-grade smartwatch and smartphone devices [28]. Using this high-dimensional multi-sensor and multidomain dataset, we sought to: (1) engineer a robust library of features using a combination of time- and frequency-domain signal processing; (2) evaluate the reliability, validity, and selectivity patterns of features for PD status; and (3) develop a machine learning model that maps engineered features onto PD status and evaluate model performance metrics across environmental and temporal contexts.

## 2. Materials and Methods

### 2.1. Study Design and Sample

Participants living with Parkinson’s disease (PD) and healthy controls (HC) were recruited to complete the multi-center (*n* = 17) WATCH-PD (Wearable Assessments in the Clinic and at Home in PD) (NCT03681015) observational study at a designated Parkinson Study Group research site. The WCG^TM^ Institutional Review Board approved the procedures used in the study, and there was full compliance with human experimentation guidelines.

Criteria for enrollment into the PD group included: (1) a diagnosis that has been clinically documented by a movement disorder specialist; (2) the participant is older than 30 years at diagnosis; (3) a disease duration of less than two years; (4) a Hoehn and Yahr stage of <3; (5) no baseline use of dopaminergic or other PD medications; and (6) no alternative Parkinsonian diagnosis. Approximately half of the PD group underwent DaTscan screening to confirm their diagnosis. Criteria for enrollment into the HC group included: (1) age-match to the PD group; (2) no previous PD diagnosis; and (3) no other significant neurologic disease. All participants provided written informed consent before study participation. The HC participants underwent clinical assessment, including MDS-UPDRS screening, to confirm a lack of Parkinsonism.

Participants completed a one-year longitudinal study comprising both traditional clinic visits (*n* = 6) and remotely monitored home visits (*n* = 24). Clinic visits were completed at baseline and in months 1, 3, 6, 9, and 12. Home visits were completed every other week. Clinical visits served to establish traditionally optimal environmental conditions for collecting performance data from the mobile assessment battery; in contrast, home visits served to evaluate whether similar levels of performance could be observed when the tests were completed remotely without direct clinical supervision. Participants completed the same mobile assessment battery in both environments.

### 2.2. Mobile Assessment Battery

Participants completed the BrainBaseline Movement Disorders mobile assessment battery (Clinical ink; Winston-Salem, NC, USA) as part of their study participation [28]. The BrainBaseline Movement Disorders mobile assessment battery was completed on a provisioned Apple smartphone (iPhone XS Max or 12 Pro; OS versions 12.1-15.1; Cupertino, CA, USA) and smartwatch (Apple Watch 4 or 5; OS versions 5.1.2-8.0) devices. Smartwatches were worn on either the most strongly affected wrist (PD group) or the non-dominant wrist (HC group). Smartphones were primarily held with both hands during the battery, apart from the Gait and Balance task, in which the phone was worn on the trunk. Brief assessment descriptions are as follows (Table 1):

- Visual Short-Term Memory (VSTM): Participants were instructed to remember four colored squares and to respond, after a brief blank display screen, as to whether a single probe color matched one of the previously remembered squares. Response accuracy was the primary outcome measure.
- Symbol Digit Modalities Test (SDMT): Participants completed a modified SDMT in which they were presented with a symbol, matched this symbol to an appropriate number within a symbol–number key, and then verbalized the appropriate number before proceeding to the next symbol. The total number of completed symbols was the primary outcome measure.
- Trails: Participants completed a digital version of the Trail-Making Test, in which they were instructed to draw lines between target objects in either numerical order (i.e., 1-2-3-4) or in alternating number-letter order (i.e., 1-A-2-B-3-C). The total time to completion was the primary outcome measure.
- Fine Motor: Participants were presented with a randomly positioned and oriented major circular sector and were instructed to drag and rotate the object to match the position and orientation of a target sector. The total number of completed objects was the primary outcome measure.
- Finger Tapping: Participants were presented with two circular target locations and instructed to rapidly tap the center of each location with their pointer and middle fingers in alternating order. The total number of taps was the primary outcome measure.
- Gait and Balance: During the Gait task, participants were instructed to walk as they normally would for 60 s. During the Balance task, participants were instructed to stand with their feet shoulder-width apart and remain motionless for 30 s. Motion data were then captured from smartphone and smartwatch sensors during the Gait and Balance tasks.
- Tremor: Tremor testing comprised Postural and Resting Tremor sub-tasks. During the Postural Tremor sub-task, the participants were seated and instructed to maintain their arms in a straight line at a 90-degree angle with respect to their body for 10 s. During the Resting Tremor sub-task, the participants were seated and instructed to maintain their arms at rest by their sides for 10 s. Motion data were captured from smartwatch sensors during the Tremor tasks.
- Phonation: Participants were instructed to speak and sustain the phoneme ‘ahh’ for as long as possible within a 15-s measurement window. Speech data were captured from the smartphone microphone and encoded in .wav format.
- Articulation: Participants were instructed to speak and repeat the phoneme sequence ‘pa-ta-ka’ as many times as possible within a 15-s measurement window. Speech data were captured from the smartphone microphone and encoded in .wav format.
- Reading: Participants were instructed to read three sentences sampled from the Harvard Sentences Bank at a rate reflecting their typical reading speed. Speech data were captured from the smartphone microphone and encoded in .wav format. In the present work, the Reading task data were excluded from the analysis.

Clinic visits were initiated by study-site personnel and self-administered by participants. For home visits, the participants received reminder notifications at 9:00 a.m. local time and, once initiated, were given 1 h to complete their self-administered scheduled activities.

### 2.3. Feature Engineering

Features were engineered from all the continuous data sources available from each assessment (see Table 1). Our library of features was modeled after previous works, extracting features from cognitive [29,30], speech [31,32], and accelerometer-based mobility assessments [33,34,35]. Details regarding the feature engineering routine are as follows.

VSTM. Two features were extracted from VSTM: mean response accuracy and working memory capacity (K).

SDMT. Two features were extracted from SDMT: total completed symbol–digit pairs and mean response accuracy.

Trails. Twelve features were extracted from Trails. The total completion time was extracted from A and B sub-tasks. Additional features were engineered from the reconstructed path drawn during each subtask: (1) total reconstructed path length; (2) root mean squared error between the reconstructed and idealized paths between target locations, and total spectral energy in the 4–10 Hz band observed within: (3) reconstructed path tracings in the vertical screen dimension; (4) reconstructed path tracings in the horizontal screen dimension; and (5) residual errors between reconstructed and idealized paths.

Fine Motor. Nineteen features were extracted from Fine Motor. The total completed with the dominant hand, the total completed with the non-dominant hand, and the ratio of the total completed with the dominant to non-dominant hands were extracted from the summary data. For each hand, additional features were engineered from raw time series data, reflecting the reconstructed position and orientation of target objects: (1) total path length; (2) ratio of the total path length to the idealized path length; (3) movement speed (pixels/millisecond); (4) total rotation; (5) the ratio of total rotation to idealized rotation; (6) rotation speed (degrees/millisecond), and total spectral energy observed within the 4–10 Hz band: (7) reconstructed position tracings in the vertical screen dimension; and (8) reconstructed position tracings in the horizontal screen dimension

Finger Tapping. Twenty-eight features were extracted from Finger Tapping. The number of alternating taps, the number of total taps, and the ratio of alternating to total taps for both dominant and non-dominant hands were extracted from the summary data. For each hand, additional features were engineered from raw time series data, reflecting tap position and duration: (1) median and inter-quartile range (IQR) of tap duration; (2) median and IQR of tap distance from the target location; (3) median and IQR of tap onset asynchrony time; (4) median and IQR of the inter-tap interval; (5) IQR of the spatial variance in vertical and horizontal screen dimensions; and (6) peak spectral frequency of the tap time series.

Tremor. Triaxial accelerometry data were generated with respect to the smartwatch reference frame. Prior to implementing feature engineering libraries on Tremor data, preprocessing routines linearly rotated the triaxial accelerometry data to triaxial gravitational acceleration data to produce a watch-independent geospatial reference frame, in which the *z*-axis forms a 90-degree angle with respect to the surface plane. Velocity and position were derived by calculating the first and second cumulative trapezoidal integrals, respectively, of the raw accelerometer signal.

Two hundred and thirty-one unique features were engineered from the Tremor tasks, yielding 462 total features. Of these, 20 univariate time-domain features were engineered separately from each accelerometer axis: (1) zero-cross rate; (2) summary statistics (median, mean, 25th percentile (Q_1_), 75th percentile (Q_3_), IQR, and the standard deviation (SD), kurtosis, skewness) of inter-cross interval time distributions; (3) SD and IQR of raw acceleration; (4) summary statistics (mean, median SD, Q_1_, Q_3_, and IQR) of integrated velocity; and (5) the total path length of acceleration, velocity, and position. Univariate time-domain analyses yielded 60 unique features.

Two multivariate time-domain features (total path length and convex hull area) were engineered separately from each unique combination of accelerometer axes (x-y, x-z, y-z, and x-y-z) for acceleration, velocity, and position. Multivariate time-domain analyses yielded 24 unique features.

Forty-nine univariate frequency-domain features were engineered separately from each accelerometer axis: (1) spectral energy within the following energy bands: 3.5–4.5 Hz, 4.5–5.5 Hz, 5.5–6.5 Hz, 6.5–7.5 Hz, 7.5–8.5 Hz, 8.5–9.5 Hz, 9.5–10.5 Hz, and 4–10 Hz; (2) spectral roll-off at 1%, 25%, 50%, 75%, and 99% percentiles; (3) Mel-frequency cepstral coefficients (MFCC) 1–16, calculated within the 0–49 Hz range; and (4) summary statistics (entropy, SD, kurtosis, skewness, and flatness) of total spectral energy (0–49 Hz), spectral energy within the 4–10 Hz band, spectral energy outside the 4–10 Hz band, and the ratio of energy from within to outside of the 4–10 Hz band. Univariate frequency-domain analyses yielded 147 unique features.

Gait and Balance. Triaxial accelerometry data were generated with respect to the smartwatch and smartphone reference frame. Prior to implementing feature engineering libraries on the Gait and Balance tasks, several preprocessing routines were performed. Triaxial accelerometry data from both devices were linearly rotated into a common geospatial reference frame, as performed on Tremor data. Smartphone and smartwatch data streams were temporally synchronized to ensure that each sample was comparable in time. Step-detection algorithms were implemented to identify periods in which foot strikes were detected in the acceleration signals. First, a 10-Hz high-pass finite impulse response (FIR) filter was applied to the raw acceleration signal, followed by the application of a 5-Hz low-pass FIR filter to the modulus of the high-pass filtered signal. Next, the second derivative of the signal was calculated and values exceeding a 15% increase in signal relative to the mean signal were counted as steps. Steps with an inter-step interval of greater than 1000 milliseconds were excluded from the analyses. Velocity and position were derived by calculating the first and second cumulative trapezoidal integral, respectively, of the raw accelerometer signal.

In total, 1053 unique features were engineered from the Gait and Balance tasks, yielding 2106 total features. The time- and frequency-dependent feature engineering routines described in Tremor were implemented on both smartphone and smartwatch data from Gait and Balance, yielding 462 unique features.

Nineteen spectral coherence features were engineered from all 15 combinations of accelerometer dimensions and device pairings: (1) peak spectral coherence frequency; (2) total spectral coherence across frequencies; and (3) spectral coherence observed at 1, 2, 3, 4, 5, 6, 7, 8, 9, 10, 15, 20, 25, 30, 35, 40, and 45 Hz. The coherence analyses yielded 285 unique features.

Fifty-one gait-related features were engineered from each triaxial dimension from each device: (1) step count; (2) step frequency; (3) step distance and summary statistics (mean, median, Q_1_, Q_3_, IQR, SD, kurtosis, skewness) features derived from the data interleaving each step: (4) inter-step interval; (5) step velocity; (6) step distance; (7) the path length of acceleration; (8) the path length of velocity; and (9) the path length of position. The gait analyses yielded 306 unique features.

Phonation and Articulation. In total, 495 unique features were engineered from the Phonation and Articulation tasks, yielding 990 total features. The total speech duration was extracted from the summary data. Voice waveforms were divided into 15 segments of equal length, and summary statistics (mean, median Q1, Q3, IQR, 1st percentile, 99th percentile, IQR_99_, SD, kurtosis, skewness, flatness, and root mean square) were derived for 38 features generated within each segment: (1) fundamental frequency; (2) harmonic-to-noise (HNR); (3) jitter, in both milliseconds and percentage; (4) shimmer, in both decibels and percentage; (5) total energy within the 50–80, 80–120, 120–200, 200–360, 360–680, 680–1500, and 1500–4000 Hz spectral energy bands; (6) the flatness, entropy, variance, skewness, and kurtosis of all spectral energies; (7) spectral roll-off at 25%, 50%, 75%, and 90% percentiles; and (8) MFCC1–16.

Evaluating Disease Relevance of Features. In an exploratory analysis, we evaluated whether our engineered features demonstrated disease or behavioral relevance by inspecting the proportion of all features that were associated with early-stage PD status. Importantly, separate feature selection routines were implemented during the machine learning modeling phase of this analysis. To enumerate how many features showed significant distributional differences between the PD and HC participants, univariate linear regression was performed on each feature independently, wherein a given feature was the dependent variable and the participant’s group (PD, HC) was a categorical independent variable (*Feature~Group [PD, HC]*). F-statistics from each feature-wise univariate test were aggregated into a distribution. We then evaluated the overall proportion of features showing significance, as well as the proportion of features within each assessment showing significance. Given the exploratory nature of this analysis, thresholds for feature-wise significance were set to *p* = 0.05 and corrections for multiple comparisons were not implemented.

### 2.4. Machine Learning Modeling

To evaluate the robustness of the machine learning models, all machine learning analyses were implemented using a Monte Carlo simulation (*n* = 100) on data sorted subject-wise into training (90% of all participants) and test (10% of all participants) sets for cross-validation.

Model Comparison. To identify the model that was most predictive of PD status, model comparison analyses were conducted using the following steps in turn: feature wrangling, feature selection, feature reduction, model development, model evaluation, and model selection.

Data wrangling was performed to combine multiple data sources into a single data frame prior to implementing the feature processing and modeling routines. Any given assessment produced a single array of unique features. Performing feature engineering routines across all sessions and participants produced an *m* × *n* matrix for each assessment, where *m* is the total number of sessions completed and *n* is the total number of features engineered for the assessment. Each feature matrix was merged into one data frame, where each row was a unique participant and session. For example, if a participant partially completed some assessments in a single session, the row for that session would be missing some features. Features generated across all sessions—both at home and in the clinic—were averaged together for each participant, producing a 1 × *n* row of features for each participant, thereby minimizing the session and temporal variance in the measurements and normalizing features to a normal distribution.

Feature selection routines were performed to reduce the dimensionality of the features used during modeling by identifying those features that best discriminated between PD and HC participants. To this end, we used univariate linear regression (as described in *Evaluating Disease Relevance of Features* in Section 2.3) and sorted the features by F-statistic value. Features (*n* = 100) demonstrating the highest F-statistic were selected for modeling and the remaining features (*n* = 3521) were excluded from further analysis. Notably, not all features showing a significant association with early-stage PD status were selected for further analysis. As will be described below, feature selection was parametrically included or omitted from the analysis routine to evaluate the impact of feature selection on model performance.

Feature reduction routines were performed to reduce the dimensionality of the features used during modeling by eliminating multicollinearity between features. To this end, we used principal component analysis using the Python scikit-learn function *decomposition.PCA*. Principal components (*n* = 10) explaining the greatest amount of variance in the input features were selected for further analysis and the remaining principal components were excluded from further analysis. A fixed number of principal components were selected to ensure that the data formats were consistent across models. As will be described below, feature reduction was parametrically included or omitted from the analysis routine to evaluate the impact of feature reduction on model performance.

To evaluate the impact of feature processing on model performance, the feature selection and feature reduction routines were parametrically manipulated. For feature selection, either a subset of features (*n* = 100) was selected for modeling (selection +), or all features were selected for modeling (selection −). For feature reduction, either principal components (*n* = 10) were extracted from the raw features and selected for modeling (reduction +) or the raw features were selected for modeling (reduction −). Consequently, each model included four possible feature-processing steps: (1) selection (+), reduction (+); (2) selection (+), reduction (−); (3) selection (−), reduction (+); and (4) selection (−), reduction (−). Prior to model development, features in both the training (90% of participants) and test (10% of participants) sets underwent one of these four processing routines. The performance of each model was compared against the context of the inclusion and exclusion of each of these feature-processing steps.

Nine unique models were developed and evaluated: logistic regression (LR), linear discriminant analysis (LDA), support vector machine (SVM), decision tree (DT), gradient boosted tree (GBT), random forest (RF), stochastic gradient descent (SGD), Gaussian naïve Bayes (GNB), and multilayer perceptron (MP). Various machine learning models were evaluated due to the lack of convergence in validated models in the current literature. Each model underwent four feature-processing routines through separate iterations, yielding 36 total unique models. Each model was developed and trained on PD training group labels while the PD test features and labels were withheld from analysis. Critically, training and test features were sorted subject-wise so that feature sets for a given participant were sorted into either the training set or the test set. The training model coefficients were saved for model evaluation.

Model evaluation was implemented by inspecting how well each model performed in predicting PD test labels. To this end, we applied the training model coefficients to the independent test set features to generate predictions of the PD test group labels. PD labels and model predictions were convolved into a 2 × 2 table, from which the model performance metrics of accuracy, sensitivity, and specificity were calculated. These procedures were implemented iteratively across all Monte Carlo simulations (*n* = 100), generating a sample of independent model prediction metrics based on a random assortment of features into training and test sets on each pass through the Monte Carlo simulation. Critically, all models were evaluated on the same training and test data across each Monte Carlo simulation. The models were trained and tested using the scikit-learn Python library.

Model selection was performed to identify the model most predictive of PD status. To this end, we used the Wilcoxon signed-rank test to compare the paired samples (*n* = 100) of model performance metrics generated by each Monte Carlo simulation iteration. In cases where multiple high-accuracy models were statistically indistinguishable from each other, the most parsimonious model was selected. Here, the most parsimonious model was defined as the least complex model requiring the fewest number of feature-processing procedures.

Cross-Environmental Predictions. We sought to determine whether the performance and sensor data generated in clinic and home environments were of sufficient comparability to support the remote screening of PD status based on home assessments alone. To this end, cross-environmental learning analyses trained models on data generated by sensors and assessments in one environment (e.g., clinic) and predicted the PD status based on data generated by the same sensors and assessments in an independent environment (e.g., at home). Prior to modeling, the features generated across home and clinic visits were averaged together, yielding two vectors of data reflecting the home and visit features for each participant. Only participants with both home and clinic vectors were submitted for analysis. During model development, the participant data were partitioned into training and test sets (or cohorts), as described above.

We used the model selected from the Model Comparison analysis and implemented the same Monte Carlo simulation methods. Model training and testing routines were conducted using a 2 (training environment: home vs. clinic visits) × 2 (testing environment: home vs. clinic visits) × 2 (model test dataset: same vs. independent cohort) study design. For example, one modeling condition would be to train the model on home data generated by the training cohort and test on clinic data generated by the training cohort (training environment = home, test environment = clinic, model test dataset = same cohort), while a separate modeling condition would be to train on home data generated by the training cohort and test on clinic data generated by the test cohort (training environment = home, test environment = clinic, model test dataset = independent cohort). Using this design, we were able to evaluate: (1) how the training environment affected classification accuracy; (2) how the testing environment affected classification accuracy; (3) how models trained and tested within the same environment performed relative to models trained and tested across environments; and (4) how the cross-environment classification accuracy differed when models were trained and tested on the same or independent cohorts across independent environments.

A repeated-measures ANOVA was used to evaluate the omnibus effects of the training environment, test environment, and model test dataset. Post hoc analyses focused on: (1) the effects of cohort, to demonstrate proof-of-principle that training and testing on the same cohort inflates classification accuracy; (2) the effects of the environment on classification accuracy by evaluating the same and independent cohort conditions separately; and (3) the generalizability of the classification results to the population by evaluating model performance metrics in the clinic training environment, home testing environment, and independent cohort conditions. Importantly, classification results from model training and testing procedures that were performed on the same data were excluded from discussion to preclude the obvious concerns of overfitting.

### 2.5. Reliability Analysis

We sought to evaluate: (1) external reliability, to assess the measurement consistency across environments by comparing performance between clinic visits, conducted within a relatively controlled and supervised environment, and home visits, conducted within a relatively uncontrolled and unsupervised environment; and (2) test-retest reliability, to assess measurement consistency across time. To this end, external reliability and test-retest reliability were assessed for each engineered feature using intraclass correlations (ICC). Thresholds for acceptable reliability coefficients can range from 0.45 to 0.98 [36]. In the current work, we selected an ICC coefficient value of 0.6 as the threshold for acceptance, representing a reliable feature for both external and test-retest reliability analyses.

ICC coefficients were derived for each feature in both external and test-retest reliability analyses. Traditionally, reliability coefficients are reported individually for each feature or measurement assessed. Given the volume (*n* = 3621) of features engineered in this work, we evaluated the sample characteristics of our feature ICC coefficients relative to our reliability threshold. Specifically, we calculated the proportion of feature ICC coefficients exceeding the reliability threshold and whether the sample of feature ICC coefficients was statistically higher than the reliability threshold. To calculate the proportion of features exceeding the reliability threshold, we enumerated the number of feature ICC coefficients greater than 0.6 as a percentage of all features within a given assessment. To determine whether the feature ICC coefficients were statistically higher than the reliability threshold, we used one-sample *t*-tests to calculate whether the sample mean of ICC coefficients was greater than 0.6. Similar analyses were performed only on those features identified as important features during the machine learning modeling procedures.

### 2.6. Cross-Platform Validation

To test the robustness of our feature engineering pipeline, we leveraged data from the mPower study [37]. Volunteer participants with or without a professional diagnosis of PD utilized iPhones (4S to 6 plus, ~65% iPhone 6 of 6 plus) to complete Gait and Balance, Verbal Phonation, and Finger Tapping assessments ad libitum.

The feature engineering routines described above were implemented here. Due to differences in assessments and task administration, preprocessing modifications were necessary to facilitate comparison between the WATCH-PD and mPower platforms. WATCH-PD Gait and Balance samples were restricted to 30 s (10 to 40 s) of the 60-s recording duration, whereas mPower data containing less than 25 s of data were excluded. Since the mPower study excluded watch measurements, Gait and Balance watch data were excluded from WATCH-PD to maintain consistency across the study data sets. In the mPower finger-tapping assessment, the non-dominant hand was not evaluated so these features were excluded from the WATCH-PD dataset. Similarly, tap duration was not measured in mPower, so it was not possible to calculate duration-dependent features such as tap-off duration, frequency, and tap duration itself. Thus, these features were also excluded from WATCH-PD for equal comparison. Finally, verbal phonation features were unchanged between studies. The reduced feature set derived from mPower and the equivalent features from WATCH-PD were submitted to the same model comparison pipeline described in the Model Comparison in Section 2.4, as described above.

### 2.7. Feature Comparison

We evaluated the predictive power of mPower features extracted using our pipeline using a voting classifier in scikit-learn/python. This approach was modeled after the ensemble approach deployed in R and described by Sieberts and colleagues [26]. Specifically, a voting classifier was constructed from elastic net, random forest, support vector machine, k-nearest neighbor, and neural network-based approaches. The classifier was trained on 80% of the data (the per-subject median of each feature) and tested on the other 20% over 50 bootstraps, and the area under the receiver operating characteristic (auROC) curve was used as an accuracy metric. For comparison, we repeated this approach on the accelerometer feature sets employed by Sieberts and colleagues.

## 3. Results

### 3.1. Study Sample and Data

PD (*n* = 82) and HC (*n* = 50) participants enrolled in the WATCH-PD (NCT03681015) study across 17 Parkinson Study Group research sites between June 2019 and December 2020 (see Section 2 for enrollment criteria). Study participants were demographically matched across groups, with the exception that PD participants were more likely to be male. MDS-UPDRS ratings were higher in PD participants for both total (PD: 35.2 ± 12.4; HC: 5.9 ± 5.3; *p* < 0.001) and Part III (PD: 24.1 ± 10.2; HC: 2.7 ± 3.5; *p* < 0.001) scores [28].

As part of the 1-year longitudinal study, participants completed the BrainBaseline Movement Disorders platform assessment application (Clinic ink; Winston-Salem, NC, USA) (Table 1) on their smart devices. Assessments were designed to assess a range of functional domains known to be impacted by PD. Participants completed the assessment application during both traditional on-site “clinic” sessions (*n* = 6), which included additional clinical evaluations described elsewhere [38], and remote visits in which participants completed the assessment application at “home” (*n* = 24). Clinic visits were completed at baseline and in months 1, 3, 6, 9, and 12. Home visits were completed every other week. Clinical visits served to establish traditionally optimal environmental conditions for collecting performance data from the mobile assessment battery; in contrast, home visits served to evaluate whether similar levels of performance could be observed when tasks were completed remotely without direct clinical supervision. Critically, participants completed the same mobile assessment battery (Table 1) in both clinic and home sessions.

The overall assessment application compliance was 79% and 70.4% for clinic and home visits, respectively, and assessment application compliance rates were similar across the study groups (*p* = 0.86). Participants completed 24,882 total assessments and the proportion of all assessments completed by the PD group was consistent across each task and was similarly consistent with the proportion of all study participants (Table 2).

### 3.2. Feature Engineering

A total of 3621 features were engineered from all platform assessments (Table 3). Time- and frequency-dependent signal processing routines were implemented to extract disease- and behavior-relevant features from the continuous sensor data generated from the assessment battery (see Section 2). Features engineered from the Trails and Fine Motor assessments were associated with the total path length of on-screen touch movement patterns, deviations between idealized and observed on-screen completion paths, and the degree of tremor-related activity in on-screen touch movement patterns. Features engineered from Finger Tapping were associated with the distributional properties of tap duration times, inter-tap onset asynchrony, inter-tap interval times, spatial variance in the tap location and distance from the target tap location, and peak tap frequency. Features engineered from Tremor were associated with the distributional properties of acceleration, velocity, acceleration zero-cross rate, the total path length of acceleration and velocity, the total area of acceleration and velocity over time, spectral activity within the tremor frequency range, spectral roll-off, and Mel-frequency cepstrum coefficients (MFCC) 1–16. Features engineered from Gait and Balance were associated with the spectral coherence between smartwatch and smartphone acceleration patterns, the distributional properties of step count, step frequency, inter-step interval times, and the total path length between steps, in addition to the same features derived from Tremor. Features engineered from the Phonation and Articulation assessments were associated with the distribution properties of pitch, harmonic-to-noise ratio, jitter, shimmer, spectral energy bands, spectral roll-off, and MFCC1–16.

**Figure 1:**
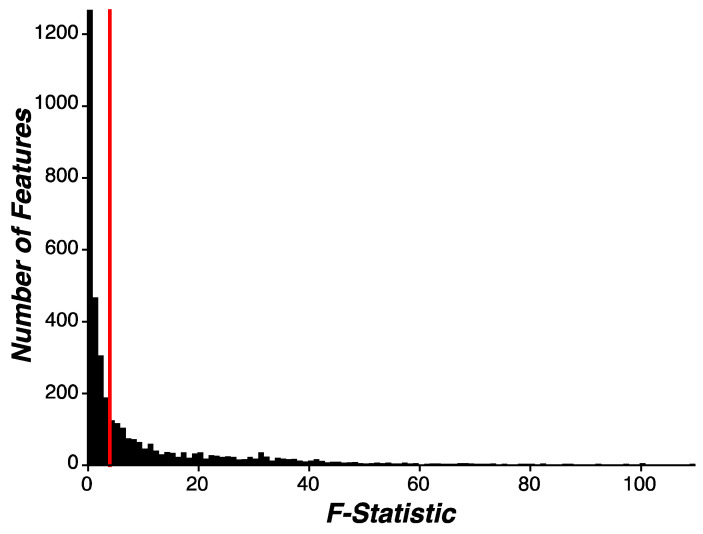
**Evaluating feature selectivity.** Linear regression was performed on each feature independently to evaluate feature selectivity for early-stage PD status. F-statistics from feature-wise linear regression were aggregated into a distribution and the threshold for significance was set to *p* < 0.05 (red line). In fact, 38.6% of all features were significantly associated with early-stage PD status. Variability in feature selectivity was observed across assessments (Table 3).

In an exploratory analysis, we evaluated whether our engineered features demonstrated disease or behavioral relevance by inspecting the proportion of all features associated with early-stage PD status. Briefly, we performed feature-wise univariate linear regression and aggregated all 3621 F-statistic values into a distribution (Figure 1; see Section 2 for more details). We found that 1398 of all feature-wise univariate linear regression tests demonstrated significance between PD and HC participants, indicating that 38.6% of all features were associated with early stage-PD status. Next, we evaluated which assessments generated features that were significantly associated with early-stage PD status at a proportion higher than chance (*p* = 5%). To this end, we grouped the F-statistics from each feature-wise univariate test by assessment and implemented the same analysis described above. We found that nearly all assessments generated features significantly associated with early-stage PD status at a proportion higher than chance, except for VSTM (Table 3). Separate routines were implemented for feature selection during the machine learning modeling analysis.

### 3.3. Machine Learning Model Comparison

To identify the optimal model for classifying PD status using features engineered from the assessment battery, a model comparison analysis was performed on data aggregated across all study visits. Briefly, we used a Monte Carlo simulation (*n* = 100) to evaluate the robustness of model performance metrics across nine machine learning models, implementing parametric manipulations of feature selection and feature reduction routines (see Section 2) via univariate linear regression and principal component analysis, respectively. For any given iteration of the Monte Carlo simulation, the same training (90% of all subjects) and test (10%) datasets were implemented across all model construction and evaluation phases, respectively, whereas the sorting of the training and test datasets was randomized across the simulation iterations using subject-wise cross-validation. Critically, feature selection and feature reduction routines were only implemented on the subject-wise training datasets.

**Figure 2:**
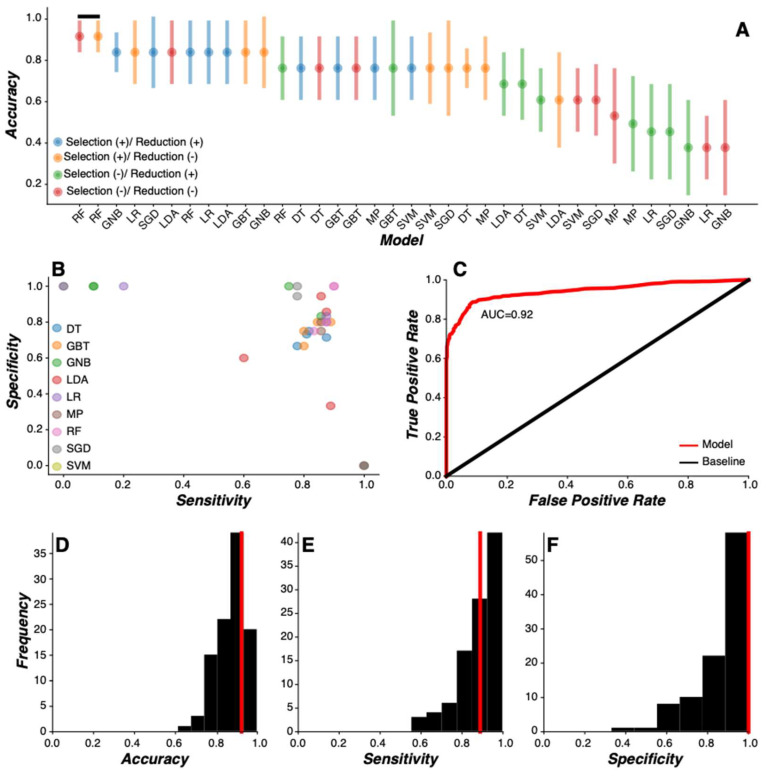
**Machine learning model comparison.** (**A**) Accuracy (median ± IQR) of machine learning model as a function of each model evaluated. Each modeling routine was preceded with (+) or without (−) feature selection and feature reduction routines (see legend). Model classification accuracy was sorted by value and all models with statistically similar classification accuracy values (Wilcoxon signed-rank test) were identified as the optimal models (black line). The random forest model without feature selection or feature reduction routines (red) was the most accurate and parsimonious model. (**B**) The sensitivity and specificity of each model were plotted as a function of each other. Four values are present for each model, representing the inclusion or exclusion of feature selection and feature reduction routines. (**C**) The receiver-operator curve (ROC) for the random forest model without feature selection or feature reduction routines across all Monte Carlo simulations (*n* = 100) showed an area under the curve (AUC) of 0.92 (IQR: 0.85–0.95) in detecting PD status. (**D**–**F**) Distribution of model classification accuracy (**D**), sensitivity (**E**), and specificity (**F**) for the random forest model without feature selection or feature reduction routines across all Monte Carlo simulations (*n* = 100). Median values for each model performance metric (red line) are denoted. (DT = decision tree; GBT = gradient boosted tree; GNB = Gaussian naïve Bayes; LDA = linear discriminant analysis; LR = logistic regression; MP = multilayer perceptron; RF = random forest; SGD = stochastic gradient descent; SVM = support vector machine).

We found the most accurate and parsimonious model of PD status to be a random forest model using raw feature values without the implementation of feature selection and reduction routines (Figure 2A,B). The receiver-operator curve (ROC) for the random forest model showed an area under the curve (AUC) of 0.92 (IQR: 0.85–0.95) in terms of detecting PD status (Figure 2C). Median model performance metrics demonstrated 92.3% accuracy (IQR= 84.6–92.3%; Figure 2D), 90.0% sensitivity (IQR = 85.7–100%; Figure 2E), and 100.0% specificity (IQR = 80–100%; Figure 2F).

**Figure 3:**
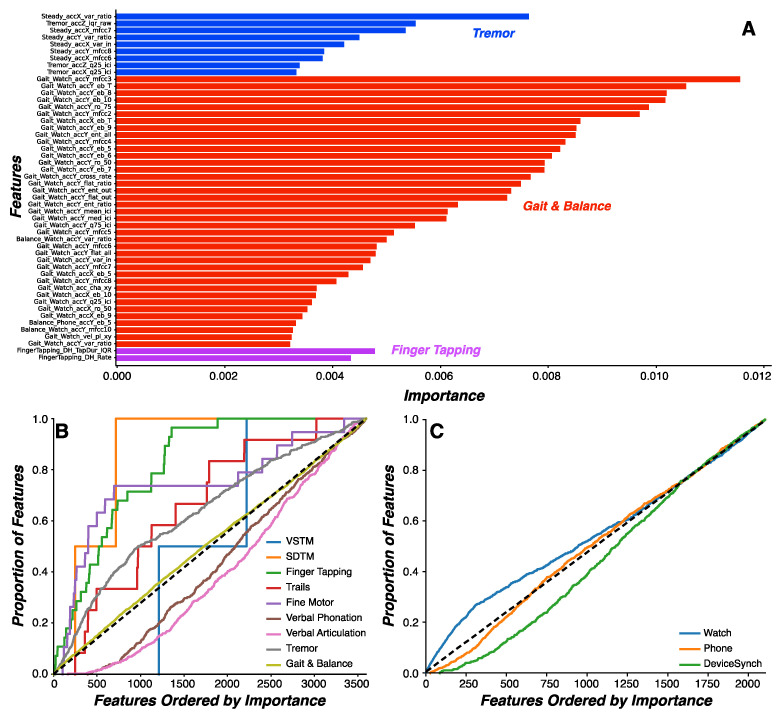
**Random forest model feature importance.** (**A**) Feature importance values (*x*-axis) for the features with the highest importance (*n* = 50; *y*-axis). Feature importance was calculated as the accumulation of the impurity decrease within each tree in the random forest model. The most important features were derived from the Finger Tapping (red), Gait and Balance (blue), and Tremor (magenta) tasks. (**B**) Cumulative probability distribution of the proportion of features represented within each assessment as a function of features ordered by importance. Features engineered from SDMT, Finger Tapping, Trails, Fine Motor, and Tremor were proportionally ranked higher in feature importance relative to chance (dotted black line). (**C**) Cumulative probability distribution of the proportion of features generated from the watch, phone, and watch–phone synchronization from the Gait and Balance task as a function of features ordered by importance. Features engineered from the watch were ranked proportionally higher in feature importance relative to features engineered from the phone and watch–phone synchronization.

Evaluating feature importance for predicting PD status in the random forest model revealed that the Gait and Balance, Tremor, and Finger Tapping tasks produced the 50 most important features (Figure 3A). Feature importance was calculated as the accumulation of the impurity decrease within each tree in the random forest model. Finger Tapping features (*n* = 3) were associated with tapping efficiency in both the dominant and non-dominant hands and variance in tap duration in the dominant hand. Gait and Balance features (*n* = 39) were derived primarily from the smartwatch during the Gait sub-task and were associated almost exclusively with the distribution around spectral activity in the Tremor (4–10 Hz) frequency band. Tremor features (*n* = 8) were derived primarily from the Resting Tremor sub-task and were similarly associated with the distribution around spectral activity in the Tremor (4–10 Hz) frequency band. Critically, these features represent the most important and selective features from the total feature set and are not representative of all selective features. Indeed, 38.6% of all 3621 features were selective for PD status, including more heuristic features such as gait metrics.

We next determined whether the relative frequencies of important features could be due to the relatively higher frequency of features generated within each assessment. The relative proportions of each task among the most important features (Finger Tapping = 6%; Gait and Balance = 78%; Tremor = 16%) were higher than the proportion of features relative to each task (Table 3). The cumulative probability distributions of features within each task as a function of feature importance revealed that the SDMT, Finger Tapping, Trails, Fine Motor, Gait and Balance, and Tremor tasks all contributed important features at a rate higher than chance (Figure 3B). Given the preponderance of watch-specific features demonstrating superior importance, we further compared feature importance between smartphone and smartwatch devices during the Gait and Balance task. Critically, the same features were derived from both the smartwatch and smartphone, supporting this comparison. The cumulative probability distributions of features generated from the watch, phone, and synchronization between the smartwatch and smartphone demonstrated that watch-specific features were generally superior in this task (Figure 3C).

We next sought to evaluate how the random forest model performed when introduced to cross-environmental manipulations.

### 3.4. Cross-Environmental Predictions

We sought to determine whether the performance and sensor data generated in clinic and home environments were of sufficient comparability to support remotely screening PD status based on home assessments alone. Using the same random forest model and approach described above, training and test datasets were parametrically constructed from the home and clinic environments, where the models were trained on clinic (or home) data and tested on both home and clinic data. Thus, we were able to compare the within- and cross-environment model predictions. To determine whether our model was generalizable to the population or restricted to the current study sample, we evaluated cross-environment predictions for data generated by the same or independent cohorts (see Section 2 for more details). Only subjects who completed sessions both at home and during clinic visits were included in the analysis (*n* = 126; PD = 78, HC = 48).

**Figure 4:**
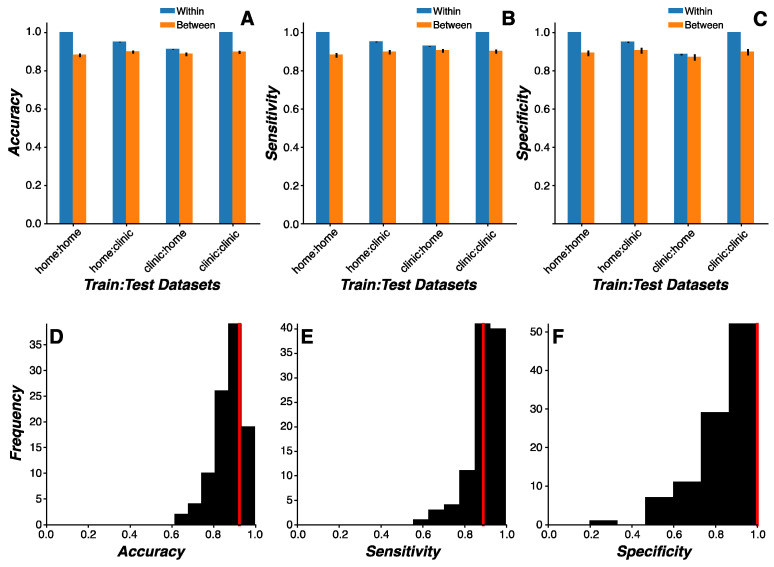
**Cross-environmental learning.** (**A**–**C**) Mean model classification accuracy (**A**), sensitivity (**B**), and specificity (**C**) for random forest model predictions as a function of training and test environmental contexts (home or clinic) (error bars represent the standard error of the mean). Model predictions were generated for either the same subjects (within; blue bars) or independent subjects (between; orange bars). Classification accuracy was higher when predictions were made on the same relative to independent subjects (*p* < 0.0001) and when models were trained on home data relative to clinic data (*p* = 0.0007). When model predictions were made on data generated by independent subjects, no difference in classification accuracy was observed across environmental contexts (*p* > 0.14). (**D**–**F**) Distribution of model classification accuracy (**D**), sensitivity (**E**), and specificity (**F**) for the random forest model trained on clinic data and tested on home data in independent subjects across all Monte Carlo simulations (*n* = 100). Median values for each model performance metric (red line) are denoted.

A repeated-measures ANOVA on model classification accuracy revealed a significant three-way interaction between the training environment (home vs. clinic), test environment (home vs. clinic), and model test datasets (same vs. independent cohorts) (F_(1,99)_ = 198.67, *p* < 0.0001) (Figure 4A–C). As expected, classification accuracy was lower when the models were tested on independent cohorts relative to the same cohort (*p* < 0.0001). Classification accuracy for models tested on the same cohort across environments (e.g., trained on home performance data and tested on clinic performance data) was better when the models were trained on home data relative to clinic data (*p* = 0.0007), which may be due to the better representation of symptom heterogeneity in the larger volume of measurements collected at home relative to clinic visits. Critically, however, the classification accuracy for models tested on independent cohorts was statistically indistinguishable across environments (*p* > 0.14), suggesting that independent validation in a separate dataset would not be contingent on the environment in which the data were collected.

The practical application of this approach would support remotely screening new patients at home before ever visiting a clinic. Using this framework, we hypothesized that models based on clinic visits using the current assessment battery would accurately predict PD status in an independent cohort of remotely monitored patients who completed the same assessment battery at home. To test this hypothesis, models were trained on clinic data from one cohort and tested on home data from a separate cohort. Median model performance metrics under these methods demonstrated 92.3% accuracy (IQR = 84.6% to 92.3%; Figure 4D), 88.9% sensitivity (IQR = 85.7% to 100.0%; Figure 4E), and 100% specificity (IQR = 77.1% to 100.0%; Figure 4F). These results suggest that BrainBaseline classifiers are robust to changes in the environment and may be reliably deployed in both home and clinical settings.

### 3.5. Cross-Platform Analysis

We next determined whether our feature engineering and machine learning approach is platform-agnostic and—as a consequence—robust across study platforms and protocols. To this end, we leveraged our feature engineering approach to extract features from the mPower study dataset [37]. These data were contributed by users of the Parkinson mPower mobile application as part of the mPower study developed by Sage Bionetworks and described in Synapse. mPower differs from WATCH-PD in several ways, including a larger sample (*n* = 1087 PD and 5581 HC), remote participation, self-reported diagnosis, and iPhone-only assessments of Gait and Balance, Finger Tapping, and Verbal Phonation.

**Figure 5:**
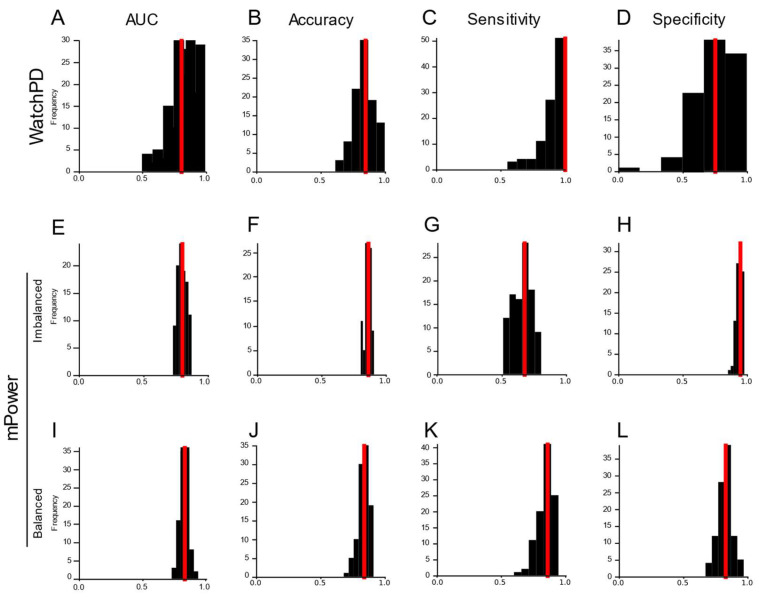
**Evaluating the mPower dataset.** Distribution of AUC (**A**,**E**,**I**), accuracy (**B**,**F**,**J**), sensitivity (**C**,**G**,**K**), and specificity (**D**,**H**,**L**) for model classification results across all Monte Carlo simulations (*n* = 100) for the reduced feature sets common to both the mPower and WATCH-PD study datasets. In the WATCH-PD reduced feature set (**A**–**D**), a random forest model without feature selection or feature reduction was the best-performing model. In both the imbalanced (**E**–**H**) and balanced (**I**–**L**) mPower feature sets, a gradient boosted tree model without feature selection or feature reduction was the best-performing model. Median values for each model performance metric (red line) are denoted.

We derived fewer features (*n* = 1271) that were common to both WATCH-PD and mPower assessments. Features were submitted using the machine learning approach described above. Analysis of the reduced WATCH-PD feature set revealed that the most accurate and parsimonious model was a random forest model without feature selection or reduction, with an AUC of 0.83 (IQR: 0.75–0.88) in detecting PD status (Figure 5A). The median model performance metrics were as follows: 84.6% accuracy (Figure 5B; IQR: 76.9–87.8%), 100% sensitivity (Figure 5C; IQR: 85.7–100%), and 75% specificity (Figure 5D; IQR: 62.5–90.6%). Analysis of the mPower feature set revealed that a gradient boosted tree model without feature selection or reduction was the best-performing model with an AUC of 0.70 (Figure 5E; IQR: 0.66–0.73), 86.2% accuracy (Figure 5F; IQR: 84.8–88.3%), 67.5% sensitivity (Figure 5G; IQR: 60.6–71.4%), and 94.6% specificity (Figure 5H; IQR: 92.5–96.0%). We hypothesized that the lower sensitivity in the mPower study was due to imbalanced group sizes (*n* = 448 PD, 1155 HC). To evaluate this hypothesis, we randomly selected a subset of HC participants that was equal in number to the PD group and performed the same model comparison analysis. Again, a gradient boosted tree model without feature selection or reduction was the most accurate and parsimonious model, achieving an AUC of 0.82 (Figure 5I; IQR: 0.79–0.84) for detecting PD status. The model performance metrics were as follows: 84.1% accuracy (Figure 5J; IQR: 81.4–86.6%), 85.7% sensitivity (Figure 5K; IQR: 81.2–88.7%), and 82.7% specificity (Figure 5L; IQR: 79.5–86.5%).

Each dataset exhibited similar patterns of feature importance. In contrast, a lower percentage of WATCH-PD features (31.5%) were significant, relative to mPower features (48% imbalanced and 39% balanced). In each dataset, a large proportion of Finger Tapping features were selective for PD status: 88.9% in WATCH-PD, 77.8% in mPower imbalanced dataset, and 77.8% in the mPower balanced dataset. Gait and Balance features showed similar levels of selectivity for PD status across WATCH-PD (39.4%), mPower imbalanced (32.1%), and mPower balanced (25.9%) datasets. By contrast, Verbal Phonation demonstrated higher levels of PD selectivity in the mPower imbalanced (62.9%) and balanced (51.9%) datasets relative to WATCH-PD (10.9%).

### 3.6. Feature Reliability

Reliable endpoints are critical to clinical trial design and patient monitoring. Here, we sought to evaluate: (1) external reliability, to assess measurement consistency across environments; and (2) test-retest reliability, to assess measurement consistency across time. To this end, we used intra-class correlations (ICC) to evaluate both measures of reliability in all engineered features. An ICC coefficient value of 0.6 was selected as the threshold for acceptable reliability [36]. Given the volume (*n* = 3621) of features that were engineered, we evaluated the sample characteristics of ICC coefficients relative to reliability thresholds by calculating the proportion of coefficients exceeding the reliability thresholds and assessing whether the sample mean of coefficients was statistically higher than the reliability thresholds.

**Figure 6:**
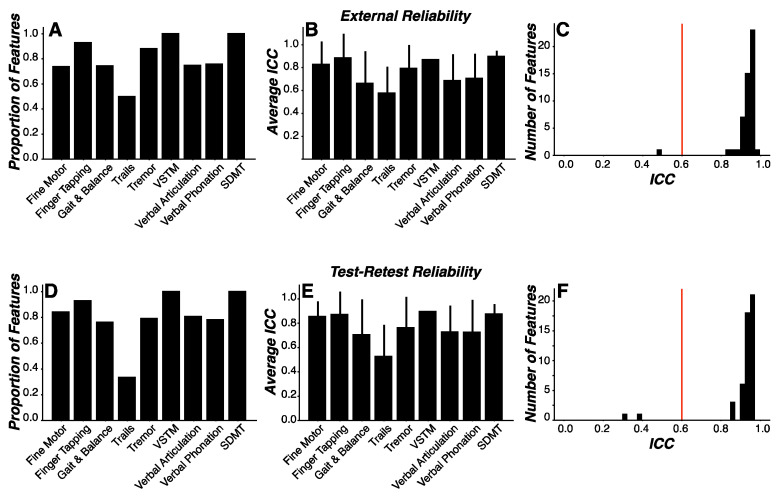
**Feature reliability.** (**A**,**B**) The external reliability of each feature was evaluated using intraclass correlations (ICC) between the clinic and home measurements. The proportion of features higher than the threshold for moderate ICC values (ICC = 0.6) (**A**) and mean ICC values (error bars represent the standard error of the mean) across all features (**B**) were evaluated for each assessment. (**C**,**D**) The test-retest reliability of each feature was evaluated using ICC between measurements across all time bins. The proportion of features higher than the threshold for moderate ICC values (ICC = 0.6) (**C**) and mean ICC values (error bars represent the standard error of the mean) across all features (**D**) were evaluated for each assessment. (**E**,**F**) Distribution of the ICC coefficients, only for features with the highest feature importance values (Figure 3A). ICC coefficients were significantly higher than the threshold (ICC = 0.6; red line) for both external reliability (*p* < 0.00001; (**E**)) and test-retest reliability (*p* < 0.00001; (**F**)) analyses.

We found that 76.4% of all features demonstrated acceptable external reliability. A preponderance of features was above the external reliability thresholds for Finger Tapping (92.9%), Gait and Balance (74.3%), and Tremor (88.1%) assessments (Figure 6A). ICC coefficients were statistically higher than the external reliability thresholds for all assessments except for SDMT and Trails (Figure 6B) (Table 4). Similarly, we found that 77.6% of all features demonstrated acceptable test-retest reliability. A preponderance of features was above the test-retest reliability thresholds for Finger Tapping (92.9%), Gait and Balance (76.3%), and Tremor (79.2%) assessments (Figure 6C). ICC coefficients were statistically higher than test-retest reliability thresholds for all assessments except VSTM, SDMT, and Trails (Figure 6D) (Table 4).

We next focused the same analysis on only those features most important for predicting PD status in the random forest model (Figure 3A). We found that 98% of these features (ICC = 0.93 ± 0.07) were above the external reliability thresholds (t_(49)_ = 33.90, *p* < 0.00001) (Figure 6E). We similarly found that 96% of these features (ICC = 0.92 ± 0.12) were above the test-retest reliability thresholds (t_(49)_ = 18.46, *p* < 0.00001) (Figure 6F).

## 4. Discussion

Consumer-grade wearable devices and sensors offer the potential for improved PD prevalence and surveillance metrics via broader access to screening tools. Here, we used a combination of feature engineering and multivariate machine learning modeling to develop a PD screening tool based on data generated from the large-scale multi-center WATCH-PD study. Our approach focused on clinically confirmed early-stage PD and HC participants who generated high-dimensional data from multiple sensors while completing a multidomain (i.e., cognitive, psychomotor, speech, and mobility) battery of assessments on consumer-grade smartwatch and smartphone devices over a one-year period. We engineered a library of low-level signal-based disease- and behavior-relevant features from the wearable device and sensor data generated by participants during their study participation. Our machine learning model comparison approach revealed a random forest model that predicted early-stage PD status with high accuracy, sensitivity, and specificity that persisted across changes in environmental contexts. Moreover, the same feature engineering and model selection approach also accurately classified later-stage PD status in an independent dataset generated on a different platform. Together, these results demonstrate the potential of consumer-grade technologies in screening early-stage PD status.

Numerous studies have evaluated wearable devices and sensors in PD symptom monitoring [17,18,19,20,21,22,23,24,25,26], though these studies focused on capturing data from a single device or sensor modality to the exclusion of capturing the multi-domain sequelae of PD. Furthermore, few among these studies have evaluated whether wearable devices and sensors can generate predictive models of PD status with direct applications to patient screening and surveillance [18,23,26]. Two studies evaluated separate cohorts of early-stage PD patients who completed voice, finger tapping, gait, balance, and memory assessments on a smartphone [18,23]. Arora and colleagues (2015) evaluated a small cohort of PD patients (*n* = 10) and non-demographically matched HC (*n* = 10) participants [18]. Using their features, they found that a random forest model of PD status achieved sensitivity and specificity of 96.2% and 96.9%, respectively. Critically, however, investigators performed record-wise rather than subject-wise cross-validation, which overestimates classification performance due to the non-independence of training and test datasets [39]. Thus, this study was limited by the small sample size, non-demographically matched study groups, and overestimated classification performance. Omberg and colleagues (2021) evaluated population-level cohorts of self-reported PD patients (*n* = 1414) and self-reported non-PD participants (*n* = 8432) participating in the unsupervised, remotely completed mPower study [23]. Using their chosen features, they found that a random forest model of self-reported PD status achieved an AUC of 0.80. In a subsequent crowdsourcing analysis of the same study’s data [26], the best crowdsourced model was a deep-learning neural network that achieved an AUC of 0.87. This study was limited, however, by its reduced control over enrollment screening and data quality, non-demographically matched study groups, and non-clinically confirmed PD status. In the current work, in contrast, we evaluated a larger demographically matched and clinically confirmed cohort of PD (*n* = 82) and HC (*n* = 50) participants, who completed visits both in the clinic and remotely while at home. Participants completed a battery of multidomain assessments that more comprehensively captured PD symptoms, including upper extremity tremor and bradykinesia using the smartwatch, relative to previous studies. Using a more appropriate machine learning methodology, including subject-wise cross-validation, our random forest model achieved superior classification performance. Importantly, our comprehensive modeling results converge with prior machine learning approaches demonstrating the superiority of tree-based models, such as random forests, in classifying PD status. Indeed, the same feature engineering and modeling approach selected a gradient boosted tree model that accurately classified participants in the mPower study. Furthermore, we sought to understand the boundary conditions of our model by evaluating its performance across environmental contexts and found that our model performs equally well when tested on clinic data collected in a controlled environment and home data remotely collected in an uncontrolled environment. Taken together, we have instrumentally extended our understanding of how device and sensor selection, assessment-to-disease mapping, and analytic methodologies are critical to tracking PD status in real-world settings.

We sought to directly compare the features generated by the watch and phone to understand whether either device is more effective at predicting PD status. To this end, our analysis focused on the same set of features being engineered from acceleration-based signals and generated concurrently from the watch and phone during the gait task. No other task generated similar data streams or features from both devices. We found that watch-derived features were probabilistically more important than phone-derived features, indicating that features related to arm swing drawn from the watch are more important than features related to gait parameters drawn from the phone in predicting PD status. This finding raises important considerations regarding the minimization of patient burden and the minimal number of devices and sensors required to capture and classify disease-relevant signals. Both the devices used in the current work captured disease-relevant information. Indeed, the exercise of mapping disease-relevant features onto multidimensional clinical scales, such as the MDS-UPDRS, demands multiple sensors and devices. Here, the watch captured those measurements relevant to tremor and arm swing, whereas the phone captured those measurements relevant to gait, speech, and finger tapping measurements. In other studies, for example, the Roche PD Mobile Application v1 used only a smartphone to evaluate Parkinsonian symptoms [20], whereas the subsequent Roche PD Mobile Application v2 added a smartwatch to better capture bradykinesia symptoms and passive mobility measurements [21]. Despite the apparent advantage of having multiple devices, a better resolution of patient symptom burden is not achieved with numerous sensors. Lonini and colleagues, for example, developed models of bradykinesia and tremor and demonstrated that a model including features from a single hand acceleration sensor performed just as well as a model including features from multiple sensors distributed across both hands, arms, and thighs [22]. Thus, a single smartphone and smartwatch set is sufficient for minimizing patient burden while maximizing Parkinsonian symptom sensitivity.

Our approach has strong applicability for developing population-wide screening tools to detect early-stage PD. Using a combination of feature engineering and machine learning model comparison routines on sensor data streams generated from a fit-for-purpose mobile PD application, we were able to construct a Random Forest model that predicted early-stage PD status with 92% accuracy. Clinical diagnostic accuracy, in comparison, ranges between 58 and 85% [8,9]. While early detection is achievable using standard clinical methodologies in the absence of remote technologies, both current practice and the existing evidence suggest that early PD is underdiagnosed, and testing demonstrates low repeatability. Our approach may, therefore, support improvements in diagnostic accuracy and reductions in the prevalence of symptomatic undiagnosed PD cases [11]. Critically, our approach aims to provide a platform for remotely screening individuals for PD and is, therefore, not intended to be a diagnostic tool. Indeed, the current platform could be a complementary tool to the MDS-UPDRS, whereby individuals identified as having PD based on the platform readout would require clinical evaluation to evaluate, diagnose, and identify the stage of their disease. Furthermore, the same platform and approach show similar promise in classifying disease status and progression in other movement disorders, including amyotrophic lateral sclerosis [40,41]. Using this remote technology platform, both physicians and patients may be better equipped to screen and monitor the transition to early-stage PD and other movement disorders, thereby minimizing the clinic and patient burden, remotely generating and accumulating diagnostic evidence over time, facilitating earlier diagnosis and access to treatment, and improving the long-term quality of life.

Our feature engineering and machine learning approach performed well on the mPower study data, an independent dataset collected from later-stage PD patients on a different platform. This study shared Gait and Balance, Verbal Phonation, and Finger Tapping assessments with WATCH-PD. Using a reduced feature set derived from the shared assessments across the platforms, we showed that a random forest model produced a classification of WATCH-PD status that was in the upper range of clinical diagnostic accuracy (84.6%). Critically, another tree-based classifier performed well on the same feature set derived from the mPower dataset (gradient boosted tree; 86.2% accuracy for the imbalanced dataset and 84.1% accuracy for the balanced dataset). While demonstrating accuracy metrics that are commensurate with clinical accuracy, models derived from the reduced feature set still underperform compared to the random forest classifier trained on the full WATCH-PD dataset (92.3%). One potential explanation for reduced accuracy is that the mPower study only collected sensor data from a smartphone, excluding measurements from smartwatches that produced features that were putatively more important than phone-based features in the WATCH-PD data, as described above. Regardless, these results suggest that our approach to feature engineering and model selection is platform-agnostic and, thus, may be applicable to a variety of existing and future studies. Standards will need to be developed, however, to account for inter-study differences in assessment implementation, sampling rate, and device type [42].

Several study limitations demand further consideration and research to better understand the potential of remote digital health technologies in supporting population-wide early-stage PD screening. (1) The sensitivities reported here may have been affected by the fact that our study comprised an enriched sample of formally diagnosed early-stage PD patients, who may have had more severe symptoms relative to non-diagnosed individuals living with PD. Indeed, we developed models of PD status using clinically confirmed patients to ensure that our model labels mirrored the ground truth diagnosis. Developing our models against traditional clinician ratings, which are demonstrably less accurate than biomarker confirmation, would have resulted in a less accurate model and a subsequently less useful tool for remotely screening PD status. To ensure that the current screening tool can detect non-diagnosed individuals living with a lower symptom burden, our approach and model must be validated in an independent study comprising a larger sample with more heterogeneous Parkinsonian symptoms. (2) Low-level signal-based features (e.g., acoustic audio features) were prioritized over high-level model-based heuristic features (e.g., speech lexical features). In PD, for example, both low-level signal-based [43] and high-level model-based heuristic [44] features have been developed for bradykinesia. Here, we prioritized low-level signal-based features to fully characterize the rhythmic activity embedded within the device- and sensor-generated signals produced by neurotypical and Parkinsonian patterns of speech and movement. Further work focused on directly comparing low-level against high-level features will be necessary from the perspectives of model predictability and explainability. (3) There are potential concerns over model overfitting, given the large volume of features (*n* = 3621) and a relatively small number of subjects (*n* = 132). These concerns, however, are mitigated by our analysis design. First, we parametrically introduced feature selection and feature reduction routines, ensuring that models constructed from saturated feature sets were compared against models constructed from feature sets with reduced dimensionality. Second, we implemented cross-validation procedures to ensure that model training and testing were performed on independent datasets, preventing any influence of overfitting during the model development of our evaluation of model performance. While it is true that some models demonstrated relatively low cross-validation accuracy, these models were equally represented across feature selection and feature reduction imputations. Thus, the selection of our best-performing model—a random forest model constructed from a saturated feature set—was not due to overfitting. Validating our model in a larger study sample with greater heterogeneity in Parkinsonian symptomatology will further seek to address these concerns. (4) The binary classification of early-stage PD was prioritized over predicting MDS-UPDRS scores because we focused on evaluating the utility of the current platform as a screening tool. Consequently, we were unable to evaluate our features and model against the clinical gold-standard MDS-UPDRS, including comparing our model against MDS-UPDRS scores, understanding the additional diagnostic value of our platform relative to clinical gold standards, and developing predictive models of PD severity and progression. (5) Our analysis was agnostic to the longitudinal design of this study. Indeed, the current work aimed to assess the potential of our approach and platform for use as a screening tool without the requirement to track changes over time. Future analyses will focus on furthering our approach by evaluating feature sensitivity and digital phenotype progression over time.

PD is the fastest-growing neurological disorder [1], impairing multiple functional domains, including cognition, motor coordination, speech, and mobility [7]. Contributors to the underestimation of the global PD burden [2,3] include low diagnostic accuracy with clinical standard measures [8,9], symptomatic undiagnosed cases [10], and challenges in identifying prodromal patients transitioning to PD [11]. Broader access to objective, repeatable, and validated remote screening assessments that capture the multidomain features of PD symptomatology stands to improve our understanding of the global PD burden and to facilitate time to treatment and care. Here, we extend our understanding of how remotely monitored consumer-grade wearable devices and sensors can contribute to better global surveillance and greater availability of PD screening. Using our comprehensive platform approach, we demonstrate that PD status can be remotely evaluated population-wide across environmental conditions with high accuracy, sensitivity, and specificity. Further validation in an independent study cohort and subsequent regulatory approval will be necessary to align this research field with the roadmap recommended by the Movement Disorders Society Task Force on Technology [45].

## Figures and Tables

**Table 1:** Summary of the mobile assessment battery.

Assessment	Functional Domain	Device(s)	Sensors	Data Elements	Sampling Interval
VSTM	Working memory	Smartphone	Phone screen	Trial type, response accuracy, and response time	Trial-by-trial
SDMT	Processing speed, working memory	Smartphone	Phone screen	Trial symbol, expected number, observed number, response accuracy, and trial duration	Trial-by-trial
Trails	Processing speed, executive function	Smartphone	Phone screen	Completion time, number of restarts, number of errors, sample time, detected screen position (x, y), nearest target, and target locations	Screen position change (23.9 ± 6.3 Hz; 8.1–54.2 Hz)
Finger Tapping	Psychomotor performance	Smartphone	Phone screen	Total taps, total alternating taps, tap time, tap location, tap duration, tap position (x, y), and tap-to-target distance	Each tap
Fine Motor	Psychomotor performance	Smartphone	Phone screen	Total completed, sample time, shape position (x, y), shape orientation, target position (x, y), and target orientation	Screen position change (10.5 ± 2.8 Hz; 1.9–23.4 Hz)
Phonation	Speech	Smartphone	Phone microphone	Speech duration, speech onset time, and raw signal (.wav)	32 kHz
Articulation	Diadochokinetic speech	Smartphone	Phone microphone	Speech duration, speech onset time, and raw signal (.wav)	32 kHz
Tremor	Postural stability and resting tremor	Smartwatch	Accelerometer, gyroscope, magnetometer, and compass	Acceleration (x, y, z), gravitational acceleration (x, y, z), orientation (roll, pitch, yaw), angular velocity (x, y, z), magnetic field (x, y, z), and heading	99.99 ± 0.5 Hz [82.5–100.8 Hz]
Gait and Balance	Gait and postural sway	Smartphone, Smartwatch	Accelerometer, gyroscope, magnetometer, and compass	Acceleration (x, y, z), gravitational acceleration (x, y, z), orientation (roll, pitch, yaw), angular velocity (x, y, z), magnetic field (x, y, z), and heading	Watch: 99.99 ± 0.5 Hz [82.5–100.8 Hz] Phone: 99.99 ± 0.5 Hz [99.2–100.8 Hz]

VSTM = visual short-term memory; SDMT = symbol digit modality test.

**Table 2:** Volume of data collection.

Assessment	Total (*n*)	PD (*n*)	HC (*n*)	% PD *	Device(s)
Participants	132	82	50	62.1	
VSTM	2820	1775	1045	62.9	Smartphone
SDMT	2817	1773	1044	62.9	Smartphone
Trails	2815	1772	1043	62.9	Smartphone
Finger Tapping	2814	1770	1044	62.9	Smartphone
Fine Motor	2812	1769	1043	62.9	Smartphone
Verbal Phonation	2820	1776	1044	63.0	Smartphone
Verbal Articulation	2813	1771	1042	63.0	Smartphone
Tremor	2605	1620	985	62.2	Smartwatch
Gait and Balance	2566	1597	969	62.2	Smartphone, Smartwatch

* Proportion of assessments generated by PD participants. VSTM = visual short-term memory; SDMT = symbol digit modality test; PD = Parkinson’s disease; HC = healthy control.

**Table 3:** Proportion of engineered features that are selective for PD status across each assessment.

Assessment	Features (*n*)	Percentage of All Features	Selectivity (%) *
VSTM	2	0.05%	0%
SDMT	2	0.05%	50%
Trails	12	0.3%	50%
Finger Tapping	28	0.8%	78.6%
Fine Motor	19	0.5%	73.7%
Verbal Phonation	495	13.7%	11.5%
Verbal Articulation	495	13.7%	14.5%
Tremor	462	12.7%	62.1%
Gait and Balance	2106	58.2%	44.1%

* Proportion of features showing significant differences between the PD and HC groups. VSTM = visual short-term memory; SDMT = symbol digit modality test.

**Table 4:** Summary of features demonstrating moderate or higher reliability.

	External Reliability		Test-Retest Reliability	
	Above Threshold (%) *	Above Threshold (*p*-Value) **	Above Threshold (%)	Above Threshold (*p*-Value)
VSTM	100	<0.00001	100	0
SDMT	100	0.073	100	0.13
Trails	50.0	0.73	33.3	0.35
Finger Tapping	92.9	<0.00001	92.9	<0.00001
Fine Motor	78.9	0.0017	84.2	<0.001
Verbal Phonation	75.8	<0.00001	78.2	<0.00001
Verbal Articulation	74.7	<0.00001	80.6	<0.00001
Tremor	88.1	<0.00001	79.2	<0.00001
Gait and Balance	74.3	<0.00001	76.3	<0.00001

* Proportion of features demonstrating moderate or higher ICC coefficients (ICC = 0.6). ** Level of significance for one-sample *t*-test between all features and the threshold for moderate reliability (ICC = 0.6). VSTM = visual short-term memory; SDMT = symbol digit modality test.

## Data Availability

The data are available to members of the Critical Path for Parkinson’s Consortium 3DT Initiative Stage 2. For those who are not a part of 3DT Stage 2, a proposal may be made to the WATCH-PD Steering Committee (jamie_adams@urmc.rochester.edu) for de-identified datasets. The code used in the study is proprietary and will not be made available. We direct readers to the data availability section on applications for data use via membership in the Critical Path for Parkinson’s Consortium 3DT Initiative or applications from qualified researchers.
